# Comparative evaluation of dentin volume removal and centralization of the root canal after shaping with the ProTaper Universal, ProTaper Gold, and One-Curve instruments using micro-CT

**DOI:** 10.34172/joddd.2021.009

**Published:** 2021-02-13

**Authors:** Hatice Yalniz, Mehrdad Koohnavard, Aysenur Oncu, Berkan Celikten, Ayse Isil Orhan, Kaan Orhan

**Affiliations:** ^1^Bezmialem Vakif University, Faculty of Dentistry, Department of Endodontics, Istanbul, Turkey; ^2^Ankara University, Faculty of Dentistry, Department of Endodontics, Ankara, Turkey; ^3^Ankara Yildirim Beyazit University, Department of Pediatric Dentistry, Ankara, Turkey; ^4^Ankara University, Faculty of Dentistry, Department of DentoMaxillofacial Radiology, Ankara, Turkey

**Keywords:** Micro-computed tomography, Nickel-titanium instruments, One-Curve, Protaper gold, ProTaper universal, transportation

## Abstract

**Background.** The main goal of our study was to assess the volume of dentin removed and transportation in root canals using ProTaper Universal (PTU), ProTaper Gold (PTG) and One-Curve (OC). Ni-Ti rotary instruments in extracted human teeth using by micro-CT.

**Methods:** Thirty human upper 1st premolar teeth with two separate root canals and sturdy, mature root tips were used in the present study. Specimens were decoronated and root length was standardized for micro CT scanning before root canal preparation done. The teeth were randomly separated into three categories (n = 10) according to the rotary NiTi system used for canal instrumentation, i.e., PTU (Dentsply, Maillefer), PTG (Dentsply, Maillefer), and OC (Micro-Mega SA). After root canal preparation, samples were scanned again on micro-CT by the same scanning parameters. Surface area, canal volume, structure model index (SMI), percentage of uninstrumented area and transportation parameters were obtained for each sample before and after micro-CT analyse.

**Results:** No significant differences between the PTG and PTU in terms of the total volume of removed dentin, surface area and percentage of uninstrumented areas were found. However, regarding to parameters above, OC showed a lower efficacy than PTG and PTU in coronal section. Regarding canal transportation, PTG and OC showed lower mean transportation values at all levels.

**Conclusion:** This paper demonstrated the root canal shaping abilities of the PTU, PTG, and OC NiTi file systems. The PTG and OC systems were associated less canal transportation and a better ability to preserve dentinal walls than PTU. There was no significance different between all rotary file systems for SMI values however, PTU and PTG showed greater canal volume and surface area change than OC file systems in coronal section.

## Introduction

Conventional endodontic treatment involves shaping, medicating, and ultimately filling the root canal system.^1,2^ The shaping step is important because the initial root canal anatomy should be shaped and enlarged according to the original contours of the canal. Moreover, the prepared root canal should have an incessantly tapered conical shape. However, these targets are not easy to get at because of the variability of root canal anatomy. Various techniques and instruments can be used to clear obstructions and overcome ledging, zipping, loss of working length, and apical transportation.^3^

Nickel titanium (NiTi) endodontic files are becoming increasingly popular because they are effective for cleaning and provide a tapered funnel shape, thus reducing chair time in endodontic treatment. However, during preparation with rotary systems, especially of curved canals, the initial root canal centralization cannot be preserved.^4^ New rotary instruments have greater flexibility and cutting capacity and have reduced the ratio of failure related to instrumentation of the root canal system.^5^ The cross-sectional design, metallic characteristic, and form of endodontic instruments are prominent factors in the transportation of the root canal.^6-8^

ProTaper Universal (PTU; Dentsply Maillefer, Ballaigues, Switzerland), produced from super elastic classic NiTi alloy, includes shaping (S1 [size 17, 0.02 taper] and S2 [size 20, 0.04 taper]) and finishing (F1 [size 20, 0.07 taper], F2 [size 25, 0.08 taper], F3 [size 30, 0.09 taper]) F4 [size 40, 0.06 taper], F5 [size 50, 0.05 taper]) files and retreatment instruments (D1, D2, and D3). ProTaper Gold (PTG; Dentsply, Maillefer) has the similar outline, configuration, and details as the PTU files, but was developed with special improved metallurgy (from Gold-wire NiTi), which makes it more flexible than PTU.^9-11^ The other type of NiTi file, One-Curve (OC; Micro-Mega SA, Besancon, France), is manufactured from heat-treated nickel-titanium alloy, called C-Wire, and offers a “controlled memory feature” that enables shaping of the entire canal with only one instrument inserted directly into the apex.^12^

Micro-computed tomography imaging (micro-CT) is an accurate and “non-invasive” method for examining a specimen before and after root canal preparation.^13^ Micro-CT imaging is recommended for analysing changes in dentin without damaging the tooth.^14^ Micro-CT imaging is frequently used due to its high accuracy, including for evaluating uninstrumented areas, the shaping potency of file systems, and untouched infected areas, all of which have a major effect on the likelihood of therapy failure after root canal preparation.^15^

The clinicians should be avoided to procedural errors characterize iatrogenic risk factors. Incomplete cleaning and preparation of the canal can lead to congestions and ridges. Once occurred, these obstacles may cause instruments to deflection, transporting the canal away from the centre of the root, until a perforation emerges. For these reasons, effective shaping, cleaning and protection of original canal form depends on some factors such as transportation, structure model index (SMI) and uninstrumented area. Canal transplantation results from the proclivity of the file to flatten and get back to its initial regular form while preparing the inclined root canal, which means removal of dentine completely from the outer surface of the curvature in the apical part of the canal.^16^ SMI should be considering for suitable preparation to root canal anatomy that includes a measurement of root convexity in three- dimensional structure.^17^

This micro-CT study was performed to compare the volume of dentin removed, transportation, SMI, and uninstrumented area in root canals between different NiTi rotary systems.

## Methods

### Selection of specimens

Thirty human upper first premolar teeth with two separate root canals and sturdy, mature root tips were used in the experiment. The necessary permission was obtained from the “Ethics Committee of Ankara University Faculty of Dentistry’’ to conduct this research. Specimens similar in coronal-apical size, which were extracted for purposes irrelevant to the present study, were collected and reserved in 0.1% thymol solution at 4°C until use. The teeth were selected based on their similarity in length (20–22 mm) and root canal curvature (<10°). Canal curvature was assessed by Schneider’s technique.^18^ Specimens were separated from their crowns perpendicular to the long axis with Endo access burs (Dentsply Maillefer) under water cooling and root length was adjusted to approximately 13 ± 1 mm from the anatomical apex for micro-CT.

### Scanning protocol before instrumentation

A high-resolution desktop micro-CT system (Skyscan 1275; Bruker, Kontich, Belgium) was chosen to scan the teeth under the following norms: 100 kVp; beam current, 100-mA; 0.5-mm Al/Cu filter; pixel size, 11.9 µm; and rotation, 0.5°. All specimens were rotated 360° within a concretion time of 5 minutes. The average scan time was around 2 hours. Other parameters are beam hardening correction and the entry of optimum contrast limits according to the manufacturer’s directions based on pre-scanning and reconstruction of the samples.

### Instrumentation of specimens

The working length was established by entering with a ‘#10 K-file’ into the root canal tip and subtracting 1 mm from the detected measurement. The teeth were randomly divided into three categories (n = 10) according to the rotary NiTi system used for canal instrumentation, i.e., PTU, PTG and OC. All procedures were performed by only one experimenter following the respective manufacturer’s suggestions. Root instrumentation was applied using by crown down technique in coronoapical direction of each tooth. To reach an identical master apical file size, the latest preparation was determined to 25 (F2) for PTU and PTG. All canals were instrumented with handpieces powered by an Endomotor (X-Smart; Dentsply Tulsa Dental, Tulsa, OK, USA). The OC system was applied by using 25 no 0.6 tapered file with 2.5 torque and 300 rpm. In each group, irrigation was performed with 2 mL of 5.25% NaOCl between use of the file. The root canals were then rinsed with 1 mL of 17% EDTA, followed by a final irrigation with 5 mL of NaOCl. Instruments were not used more than once to avoid breakage. After root canal preparations done, all samples were rescanned using micro-CT with the same parameters as used before instrumentation.

### Image analysis of micro-CT

**Figure 1:**
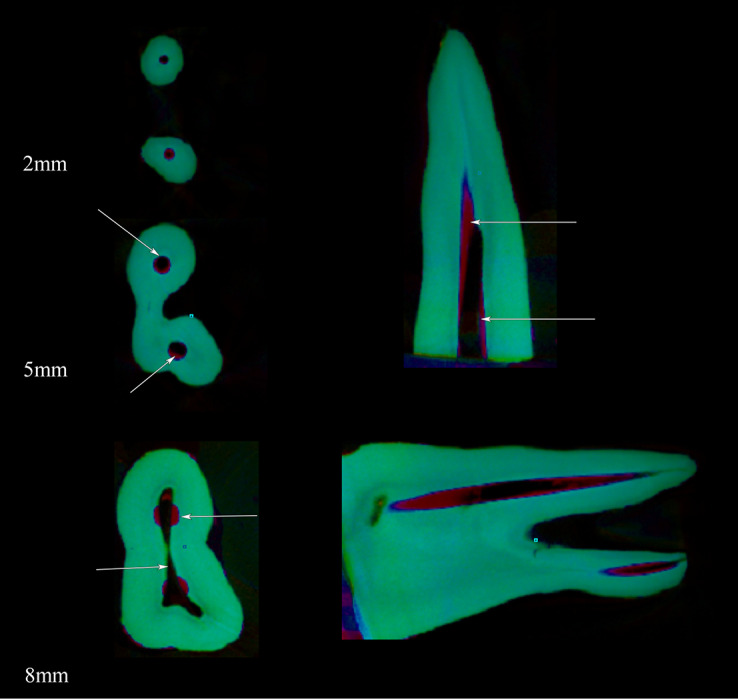
3D and cross-sectional reconstructions of premolars before and after instrumentation for 0-4, 4-8 and 8-12 mm of the root canal Note that Blue: uninstrumented areas, Red: instrumented areas Black: root canal.

NRecon software (version 1.6.10.4; SkyScan, Kontich, Belgium) and CtAn (version 1.17.7.2; SkyScan) were used for visualization and scalar analysis of the samples. Using the mentioned software, the images acquired by the scanner were reconstructed to demonstrate 2D slices of the roots.^19^ The pre- and post-instrumentation images were overlapped using the DataViewer software (version 1.5.6.2; Bruker). The superimposed datasets were delivered to CTAn software (version 1.17.7.2; Bruker) to calculate the surface area, canal volume, SMI and uninstrumented area. The reconstructed images were further processed in Skyscan CTVox (version 3.3.0; SkyScan) for visualization. SMI and dimension of the canals were also evaluated by using with triangulated data. SMI was used to describe trabecular bone with a structure as its plate or cylindrical-like geometry, which ranges from 0 (an ideal plates) to 3 (an ideal cylinder). The volume examined in each root extended from the apex to the coronal region. Three cross-sectional planes (apical, middle and coronal third; 0–4, 4–8, and 8–12 mm, respectively) were evaluated (Figure 1). The pre- and post-instrumented shortest distances from the side of the canal to the circumstance in all the roots were measured in the mesial and distal directions using the DataViewer software. Canal transportation was calculated as described previously.^20^

### Volume and area measurements

**Figure 2:**
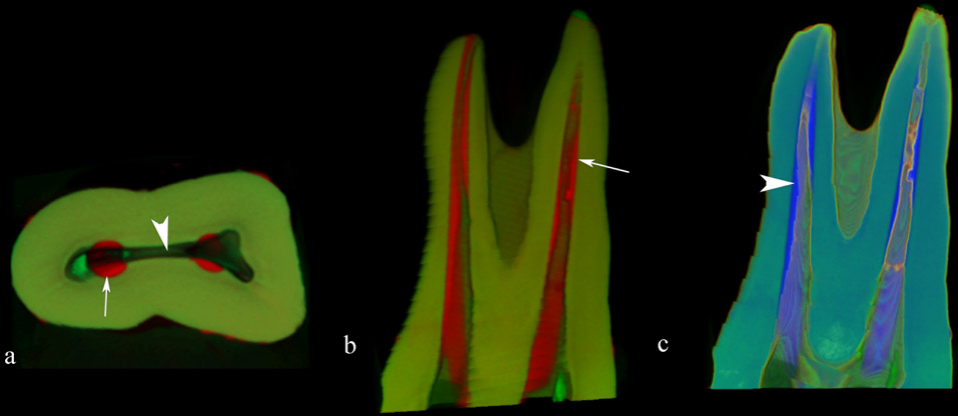
Pre- and post-instrumentation representations of root canals (**a**) axial image showing root canal pre-instrumentation (arrowhead), post instrumentation (red,arrow), (**b**) coronal image arrow indicates the canal after insturementation, (**c**) Arrowhead shows uninstrumentated areas.

Changes in volume and surface area were determined by extracting the pre and post instrumentation values using CTAn (version 1.17.7.2; Bruker). Three-dimensional (3D) surface representations were also prepared from the micro-CT images (Figure 2). The software allows the user to “sculpt out” the desired volume from the 3D structure, and to remove unwanted voxels by regulating the brilliance and opacity before calculating the recent pulp volume. The percentage of uninstrumented areas was calculated based on the ratio among the quantity of stable voxels and the total number of surfaces voxels.^21^

### Image evaluation

An examiner with 7 years of micro-CT experience assessed all images and measurements. Calculations were checked two-step by the same examiner, and the mean values of all measurements were used for statistical analysis. The examiner also performed the imaging study twice, with an interval of 1 month, so that intraobserver variability could be evaluated.

### Statistical analysis and examiner reliability

The following parameters were obtained for each sample before and after micro-CT analyses:

- Canal volume (mm^3^)
- Surface area (mm^2^)
- SMI
- Percentage of uninstrumented area
- Canal transportation

Each parameter was evaluated in the apical, middle and coronal thirds of the root canal.

Statistical analyses were performed by using SPSS software (ver. 20.0.1; SPSS Inc., Chicago, IL, USA). To evaluate intraexaminer reliability, the Wilcoxon matched-pairs signed-rank test was used for recurrent measurements. The normality of the data distribution was examined using the Kolmogorov–Smirnov test, while homogeneity was tested using Levene’ test. The data showed a normal distribution. Changes in canal volume and surface area were examined by repeated-measures ANOVA. One-way ANOVA was used for comparison among the files used in the root canals and post-hoc analyses were performed with Tukey’s and Bonferroni tests, respectively. In all analyses, *P* < 0.05 was taken to indicate statistical significance.

## Results

### Intraexaminer reliability

No significant intraobserver differences over repeated evaluations and measurements were seen, for either observer (*P* > 0.05). The overall intraobserver reliability was 90.2%. All measurements taken by both observers were found to be highly reproducible, and there were no significant differences between the two measurements for each observer (*P* > 0.05).

There were no significant differences between PTG and PTU in terms of the total volume of removed dentin or surface area (*P* > 0.05, Table 1), but OC showed significant differences in these parameters, as well as lower efficacy than PTG and PTU in coronal sections (*P* < 0.05, Table 1).For all of the NiTi rotary systems used in this study, the volumetric increase was higher in the apical third of the canal than in the other two thirds (*P* < 0.05, Table 1). There was no significant difference in SMI or percentage of uninstrumented area between the groups (*P* > 0.05, Table 1).

**Table 1:** Canal volume, Surface area, SMI, Uninstrumented area, Mean Transportation (± standard deviation) of upper first premolars after preparation with PTU (n=10), PTG (n=10) and OC (n=10)

**Ni-Ti systems**		**Canal volume (mm**^3^**)**			**Surface area (mm**^2^**)**			**SMI**			**Transportation (mean+SD, µm)**	**Uninstrumented area (%)**	
		**Pre-instrumentation**	**Post-instrumentation**	**%∆**	**Pre-instrumentation**	**Post-instrumentation**	**%∆**	**Pre-instrumentation**	**Post-instrumentation**	**%∆**			
PTU	0-4 mm	0.17±0.09	0.53±0.12	211.76	3.26±0.07	6.29±0.11	92.94	2.23±0.14	2.37±0.05	6.27	0.17±0.010^1^	For all thirds	50.94
	4-8 mm	0.59±0.11	1.64±0.16	177.96	5.28±0.21	9.18±0.27	73.86	2.27±0.21	2.49±0.03	9.69	0.15±0.016^3^		
	8-12 mm	1.22±0.17	3.05±0.33^a^	150.00^a^	7.76±0.31	13.03±0.25^c^	67.91^c^	2.36±0.33	2.68±0.08	13.55	0.10±0.012^5^		
PTG	0-4 mm	0.18±0.05	0.55±0.09	205.55	3.29±0.11	6.18±0.05	87.84	2.13±0.11	2.26±0.07	6.10	0.09±0.009^2^	For all thirds	46.83
	4-8 mm	0.58±0.14	1.59±0.18	174.13	5.31±0.15	9.01±0.19	69.67	2.34±0.16	2.55±0.05	8.97	0.08±0.012^4^		
	8-12 mm	1.25±0.18	3.04±0.34^a^	143.20^a^	7.55±0.29	12.17±0.33^c^	61.19^c^	2.41±0.29	2.71±0.11	12.44	0.05±0.018^6^		
OC	0-4 mm	0.20±0.07	0.58±0.08	190.00	3.17±0.09	5.84±0.07	84.22	2.11±0.15	2.25±0.09	6.63	0.09±0.001^2^	For all thirds	47.07
	4-8 mm	0.55±0.16	1.54±0.25	180.00	5.32±0.22	8.86±0.16	66.54	2.21±0.20	2.42±0.05	9.50	0.07±0.015^4^		
	8-12 mm	1.28±0.19	2.57±0.39^b^	100.78^b^	8.25±0.34	11.56±0.23^d^	40.12^d^	2.43±0.27	2.70±0.12	11.11	0.06±0.023^6^		

Superior different letters and numbers indicate statistical significance (p<0.05), while same superior letters and numbers show no statistical significance within groups (p˃0.05).

The highest canal transportation values were observed in the PTU group; at all levels, the values were significantly greater than those in the PTG and OC groups (*P* < 0.05).

## Discussion

Mechanical preparation is one of the most considerable procedures in endodontic treatment. Instrumentation must be adapted to the unique root canal anatomy of each case. The root canal system cannot be cleared of microorganisms and filling of the root canal cannot be done unless the canal is prepared adequately.^22,23^ The original root canal anatomy should also be preserved. This micro-CT study was performed to evaluate the effects of three rotary systems (PTU, PTG, and OC) on root canal geometry. Micro-CT is a precise, reliable, and repeatable non- destructive method that allows comparison of high-resolution pre and post instrumentation images.^14^

In this study, the total volume of removed dentin and surface area were similar between PTG and PTU (*P* > 0.05), but PTG and PTU were more efficient than OC in the coronal section for both parameters (*P* < 0.05). PTG was reported previously to remove an excessive volume of dentin in comparison to the 2 shape and Wave-One Gold instruments.^22^ Although PTU and PTG have the same geometries, PTG was developed with proprietary advanced metallurgy and heat treatment for greater flexibility and improved fatigue resistance. PTG and PTU showed similar dentin removal performance.

OC is a single-use rotary file made of heat-treated (C-Wire) NiTi alloy that allows shaping of the entire canal with a single instrument. The OC instrument has a variable cross section along the blade for better centering and cutting efficiency.^12^ Flexibility has been shown to be important to reduce transportation during root canal preparation,^23^ similar to our findings. Many studies have shown that more flexible instruments improve centralization and reduce transportation during root canal preparation.^24,25^ The PTG system was shown previously to improve centralization in the curved portion relative to the PTU system.^26,27^ In the present study, PTG and OC showed better results than PTU with regard to canal transportation. OC was less efficient in terms of the volume of dentin removed compared to PTU and PTG in coronal sections. This could be due to the sequence of PTG and PTU files SX > S1 > S2 > F1 > F2 (25/0.08) used in circumferential rasping motion, and to the convex triangular cross-section of the PTG and PTU files.^18^ Our findings were supported by previous studies comparing the canal transportation associated with heated-treated NiTi systems and those made of conventional NiTi.^9,26,28,29^

Previous studies reported that a larger proportion of uninstrumented area in the canal surface promotes the survival of microorganisms,^30-32^ which could be a risk factor for the development of apical periodontitis. Jeon et al^30^ and Hülsmann et al^31^ reported that exactly cleaning of all internal walls, and removal of canal filling materials, cannot be achieved using manual and rotary preparation methods, which usually leave areas of the canal wall uninstrumented (leading to penetration of the dentinal tubules by microorganisms^29^). Naturally, as the canal has not been instrumented in these areas, the infected inner layer of dentin will remain. Instruments with greater taper will be able to prepare a larger surface area of canal walls.^33^ In the present study, there was no significant difference among the files in terms of the size of the uninstrumented area (*P* > 0.05).

The SMI, as a measure of surface convexity, is useful for evaluating the 3D structure of an object, such as its plate- or cylinder-like geometry. Specimens with the same volume density but different architecture can be distinguished based on their SMI. The SMI ranges between 0 and 3, with 0 corresponding to an ideal plate and 3 to an ideal cylinder. This morphological criterion helps to measure the 3-dimensional characteristic model of an object. The opportunity has been given to create tools that allow more realistic representation of structures by using 3D voxel-based data sets. Some algorithms can describe whether a structure is plate- or rod-like, thus providing information about the size, shape, and geometry of local morphometric features, and improving understanding of the root canal.^34^ In this study, the mean post-instrumentation SMI values were 2.51 for PTU, 2.50 for PTG, and 2.45 for OC; the differences were not significant (*P* > 0.05). These values indicated that the root canal had a round geometry after instrumentation using these files, with an apical size of 25.

## Conclusion

This study demonstrated the root canal shaping abilities of the PTU, PTG, and OC NiTi file systems. The PTG and OC systems had less canal transportation and better ability to preserve dentinal walls than PTU. There was no significance different between all rotary file systems for SMI values however, PTU and PTG showed greater canal volume and surface area change than OC file systems in coronal section.

## Authors’ Contributions

AO and MK were responsible for the experiment design and performed the experiments in fulfillment of requirements for a degree, and wrote the manuscript. HY was responsible for the experiment design and contributed to the discussion. Data entry and statistical analyses were carried out by KO and AIO. All images were evaluated and measured by KO. BC conceived the idea, hypothesis, and the experiment design. All authors have read and approved the final manuscript.

## Funding

This study was conducted with the resources of the authors.

## Competing Interests

The authors declare no conflicts of interest related to the publication of this work.

## Ethics Approval

This study was approved by the Ethics Committee of Ankara University Faculty of Dentistry.
